# Supplementary material for: Outer Membrane Vesicles Derived from Klebsiella pneumoniae Are a Driving Force for Horizontal Gene Transfer
Source: Int J Mol Sci. 2021 Aug 13;22(16):8732. doi: 10.3390/ijms22168732 (PMC8395779; doi:10.3390/ijms22168732)
Supplement: Supplementary file 1 [file ijms-22-08732-s001.zip › ijms-1341899-supplementary.pdf]

International Journal of
Molecular Sciences

MDPI

Article

Outer Membrane Vesicles Derived from *Klebsiella pneumoniae* Are a Driving Force for Horizontal Gene Transfer

Federica Dell'Annunziata [1], Carmela Dell'Aversana [2,3], Nunzianna Doti [4], Giuliana Donadio [5], Fabrizio Dal Piaz [5], Viviana Izzo [5], Anna De Filippis [1], Marilena Galdiero [1], Lucia Altucci [3], Giovanni Boccia [5], Massimiliano Galdiero [1], Veronica Folliero [1,]* and Gianluigi Franci [5,]*

[1] Department of Experimental Medicine, University of Campania Luigi Vanvitelli, Naples, 80138, Italy; federica.dellannunziata@unicampania.it (F.D.); anna.defilippis@unicampania.it (A.D.F.); marilena.galdiero@unicampania.it (M.G.); massimiliano.galdiero@unicampania.it (M.G.)
[2] Institute Experimental Endocrinology and Oncology "Gaetano Salvatore" (IEOS)-CNR, Naples, 80131, Italy; carmela.dellaversana@cnr.it
[3] Department of Precision Medicine, University of Campania Luigi Vanvitelli, Naples, 80138, Italy; lucia.altucci@unicampania.it
[4] Institute of Biostructures and Bioimaging (IBB), CNR, Naples, 80145, Italy; nunzia.doti@gmail.com
[5] Department of Medicine, Surgery and Dentistry Scuola Medica Salernitana, University of Salerno, Salerno,84081, Italy; gdonadio@unisa.it (G.D.); fdalpiaz@unisa.it (F.D.P.); vizzo@unisa.it (V.I.); gboccia@unisa.it (G.B.)
* Correspondence: veronica.folliero@unicampania.it (V.F.); gfranci@unisa.it (G.F.)

Table S1. Protein profile commonly present in OMVs purified from *K. pneumoniae* strains.

Protein	Mass (Da)	Score	Molecular Function
Enolase	45,578	2050	Lyase
Outer membrane protein A	37,152	1501	Porin
Phosphoglycerate kinase	41,237	1449	Kinase, transferase
Outer membrane porin C	39,639	1298	Porin
Glyceraldehyde-3-phosphate dehydrogenase	32,457	1265	Oxidoreductase
Chaperone protein DnaK	69,123	741	Chaperone
Succinate - CoA ligase	41,762	501	Ligase
ATP synthase	53,363	394	ATP synthesis
Tol-Pal system protein TolB	45,767	365	Complex binding
Nucleoside-specific channel-forming protein Tsx	33,846	348	Porin
6-phosphogluconate dehydrogenase, decarboxylase	51,410	293	Oxidoreductase
Serine hydroxymethyltransferase	45,596	204	Transferase
Maltoporin	47,889	202	Porin
Polyribonucleotide nucleotidyltransferase	76,855	192	Transferase
Malate dehydrogenase	32,549	180	Oxidoreductase
Peptidase	46,475	146	Hydrolase,
Adenylosuccinate synthetase	47,334	142	Ligase
Penicillin-binding protein activator LpoA	75,265	140	hydrolase activity
Tyrosine--tRNA ligase	48,050	140	Aminoacyl-tRNA synthetase
Threonine--tRNA ligase	74,575	140	Aminoacyl-tRNA synthetase
Aspartate--tRNA ligase	66,658	132	Aminoacyl-tRNA synthetase
Catalase-peroxidase	79,031	123	Peroxidase
Arginine--tRNA ligase	64,430	117	Aminoacyl-tRNA synthetase
Methionine--tRNA ligase	76,546	114	Aminoacyl-tRNA synthetase
Phosphopentomutase	44,597	113	Isomerase
Uridine phosphorylase	27,188	107	Glycosyltransferase

Glucokinase	34,756	106	Kinase, transferase
Trigger factor	48,083	106	Chaperone
UDP-glucose 4-epimerase	15,261	103	Isomerase
Glutamine-tRNA ligase	64,199	98	Aminoacyl-tRNA synthetase
2-dehydro-3-deoxyphosphooctonate aldolase	31,033	97	Transferase
30S ribosomal protein S4	23,545	95	Ribonucleoprotein
Aminomethyltransferase	39,904	88	Aminotransferase
Elongation factor Ts	30,545	78	Elongation factor
Protein GrpE	21,707	69	Chaperone
Proline--tRNA ligase	63,541	71	Aminoacyl-tRNA synthetase
ATP-dependent 6-phosphofructokinase	35,304	66	Kinase, transferase
Glucose-1-phosphate adenylyltransferase	49,003	62	Nucleotidyltransferase
Erythronate-4-phosphate dehydrogenase	41,497	61	Oxidoreductase
Histidine ammonia-lyase	54,169	60	Lyase
ATP synthase gamma chain	31,607	60	ATP binding
Glycerol kinase	56,378	53	Kinase, transferase
Urocanate hydratase	61,979	53	Lyase
3-phosphoshikimate 1-carboxyvinyltransferase	46,287	52	Transferase
Glucosamine-6-phosphate deaminase	29,822	50	Hydrolase
Maltose/maltodextrin-binding periplasmic protein	43,109	47	Maltose binding
Pyrimidine-specific ribonucleoside hydrolase RihA	34,152	45	Glycosidase
4-hydroxy-tetrahydrodipicolinate synthase	31,427	42	Lyase
Peptide chain release factor 2	41,292	38	Translational termination
Phosphoglucosamine mutase	48,160	35	Isomerase
Deoxyribose-phosphate aldolase	27,923	35	Lyase
L-threonine 3-dehydrogenase	37,559	34	Oxidoreductase
Elongation factor P-like protein	21,556	33	translation elongation factor activity
Xaa-Pro dipeptidase	50,321	26	Protease
GTP cyclohydrolase 1	25,019	15	Hydrolase

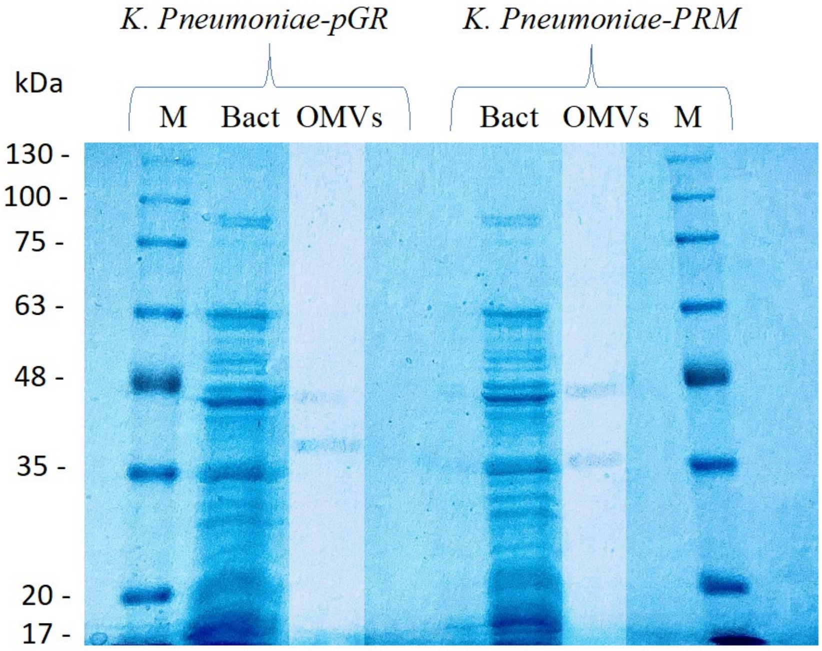

Figure S1. Coomassie-stained SDS-PAGE (10%) protein profiles of *K. pneumonia*-pGR, *K. pneumoniae*-PRM and relative OMVs. Molecular mass marker (MW) is expressed in kilodaltons (kDa).

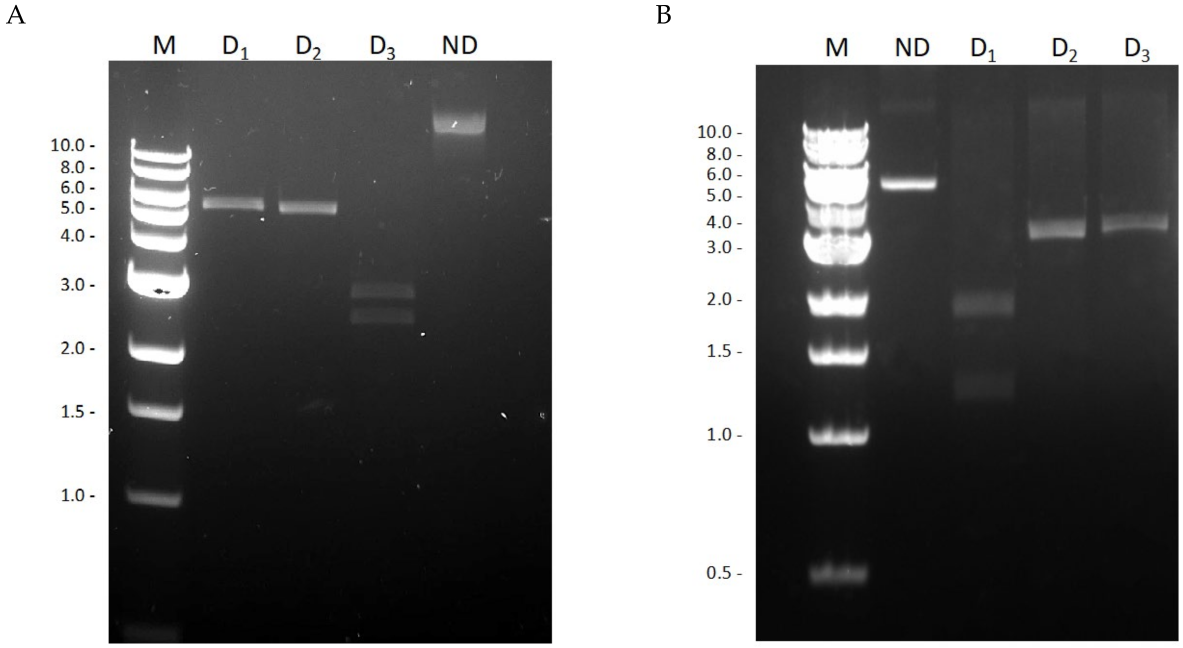

Figure S2. PGR (**A**) and PRM (**B**) plasmids enzymatic digestion. PGR and PRM plasmids were digested, in accordance with the manufacturer's instructions. Briefly, pGR (1µg) was digested using 1U of EcoRI and PvuI, respectively, in a final reaction volume of 20 µL. PRM (1µg) was digested using 1U of BglII and SacII, respectively, in a final volume of 20 µL. The samples were incubated at 37 ° C for 30 minutes and subsequently the product was visualized through 1% agarose gel. The enzymes were purchased from Promega (Madison, Wisconsin, USA). In the figure 2A was shown the pGR digestion: D_1: pGR digested with EcoRI (5047bp); D_2: pGR digested with PvuI (5047bp); D_3: pGR digested with EcoRI (2699bp) and PvuI (2348bp); ND: undigested plasmid. The figure 2B showed the PRM digestion: ND: undigested plasmid; D_1: pGR digested with BglII (1801bp) and SacII (1164bp); D_2: PRM digested with BglII (2965bp); D_3: PRM digested with SacII (2965bp).

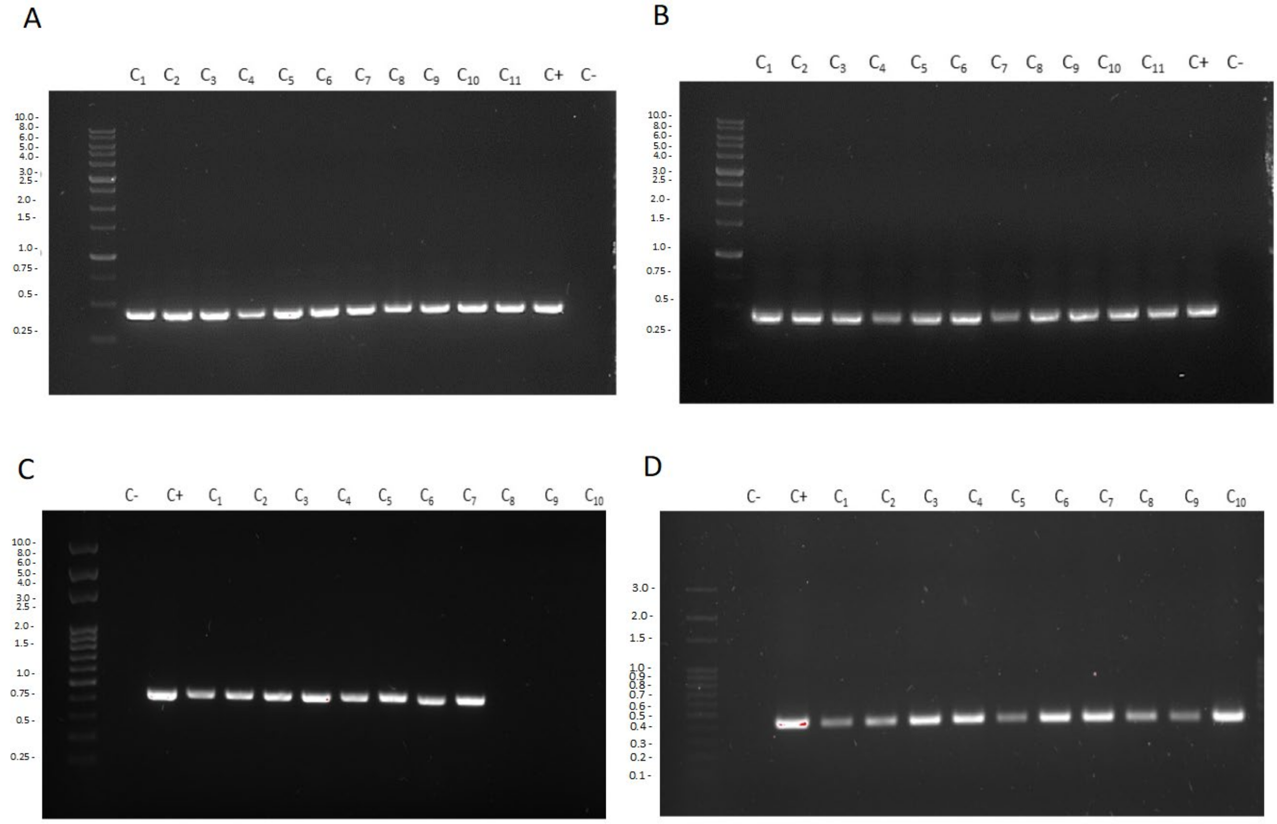

Figure S3. Colony-PCR from *E. coli* (A), *B. cepacia* (B), *P. aeruginosa* (C) and *S. enterica* recipient cells treated with *K. pneumoniae* pGR-OMVs. DNA gel showed PCR products with expected lengths: ß-lactamase product~ 424 bp ($C_{1\text{–}11}$), pGR plasmid (C_{+}), control water (C_{-}).
